# FAK-ERK activation in cell/matrix adhesion induced by the loss of apolipoprotein E stimulates the malignant progression of ovarian cancer

**DOI:** 10.1186/s13046-018-0696-4

**Published:** 2018-02-20

**Authors:** Huiling Lai, Xuejiao Zhao, Yu Qin, Yi Ding, Ruqi Chen, Guannan Li, Marilyne Labrie, Zhiyong Ding, Jianfeng Zhou, Junbo Hu, Ding Ma, Yong Fang, Qinglei Gao

**Affiliations:** 1 Cancer Biology Research Center (Key laboratory of the ministry of education), Tongji Hospital, Tongji Medical College, Huazhong University of Science and Technology, No.1095 Jie Fang Avenue, Hankou, Wuhan, 430030 People’s Republic of China; 2 Department of Systems Biology, University of Texas MD Anderson Cancer Center, TX77030, Houston, USA

**Keywords:** Apolipoprotein E, Extracellular matrix, Ovarian cancer, Tumor progression, Adhesion

## Abstract

**Background:**

Extracellular matrix (ECM) is a mediator of tumor progression. However, whether the alterations of the intraperitoneal ECM prior to tumor establishment affects the malignant progression of ovarian cancer remains elusive.

**Methods:**

Apolipoprotein (*ApoE*) knock-out mice was used to analyze the intraperitoneal ECM alterations by quantification of the major components of ECM. ID8 cells were implanted in vivo to generate allografts and human ovarian cancer cell lines were characterized in vitro to assess the effects of ECM alterations on the malignant progression of ovarian cancer. Adhesion assay, immunochemistry, cytokines profile, proliferation assay, transwell invasion assay and western blot were used to determine the malignant phenotype of ovarian cancer cells.

**Results:**

*ApoE* loss induced increased ECM deposition, which stimulated the adhesions of ovarian cancer cells. The adhesion-mediated focal adhesion kinase (FAK) signaling enhanced the invasive behaviors of ovarian cancer cells through activation of a ERK-MMP linkage. This ECM-induced signaling cascade was further confirmed in human ovarian cancer cell lines in vitro. Furthermore, reversal of the ECM accumulation with BAPN or abrogation of adhesion-induced ERK activation in ovarian cancer cells with MEK inhibitors (MEKi) was found to effectively delay ovarian cancer progression.

**Conclusions:**

These findings identify the FAK-ERK activation in cell/matrix adhesion in the malignant progression of ovarian cancer and the efficiency of BAPN or MEKi for tumor suppression, providing an impetus for further studies to explore the possibility of new anticancer therapeutic combinations.

**Electronic supplementary material:**

The online version of this article (10.1186/s13046-018-0696-4) contains supplementary material, which is available to authorized users.

## Background

Ovarian cancer is the deadliest gynecological cancer, with an overall 5-year survival rate of less than 40% [1]. Because most patients do not have symptoms at early stages, over 70% of patients have their cancer detected at an advanced stage, which makes it harder to treat [2]. Current first-line treatment strategies for advanced ovarian cancer include surgery and chemotherapy. Unfortunately, although most of the patients respond to the first treatment, a recurrence of the disease is frequently observed.

The ECM is a complex network of proteins and polysaccharides that are produced and secreted by surrounding cells. It is composed of a large variety of molecules such as metalloprotease, glycoproteins and growth factors. The main function of the ECM is to provide a structural support by maintaining the scaffold, shape and dimensions of complex tissues [3]. Differential expression of individual components of the ECM underpins its specific functions. In the case of cancer, ECM reprogramming contributes to tumorigenesis, metastasis, recurrence and resistance to chemotherapy [4]. These effects occur due to the disruption of ECM synthesis and secretion, as well as alterations of matrix-remodeling enzymes including lysyl oxidase (LOX) and MMPs [5]. Increased ECM deposition induces changes in the biochemical and biomechanical properties of the ECM, promotes the invasion of cancer cells, and renders a pro-tumoral microenvironment for cancer cells dissemination. For these reasons, targeting the key ECM components or their related modifying enzymes emerges as a potential therapeutic opportunity [6]. In the case of ovarian cancer, several studies suggest that the ECM is a mediator of tumor progression. Indeed, in serous ovarian cancer, TGFβ signaling regulates a collagen-remodeling gene signature associated with metastasis and poor survival [7]. Another report showed that TGFβ1 secreted by ovarian cancer cells drove early metastasis by enhancing fibronectin secretion by mesothelial cells [8]. These studies strongly suggest that ovarian cancer progression is dependent on the ECM composition.

ApoE, an essential constituent of the plasma lipoproteins, plays a major role in lipid metabolism. Different expression levels of specific isoforms (*E2*, *E3*, and *E4*) have been associated with the pathogenesis of cardiovascular diseases, neurodegenerative disorders and cancer [9]. In cancer, ApoE expression has an inverse correlation with the stage, treatment response and prognosis [10–12]. Contradictorily, ApoE suppresses metastasis by reducing the invasive behavior of cancer cells and by inhibiting endothelial cells recruitment in melanoma [13]. In ovarian cancer, overexpression of ApoE has been observed in clinical specimens, including serum, primary tumors and metastases, and ApoE is required for cell proliferation and survival [14, 15]. Also, cancer cells displayed high level of ApoE in a mouse model of high grade serous ovarian carcinoma [16]. Taken together, these studies indicate that ApoE can alter the tumorigenesis and tumor progression of various cancers and might have a role in ovarian cancer progression.

In a well-designed study, ApoE/ApoE-high density lipoprotein (HDL) alleviates arterial stiffening by suppressing ECM synthesis and secretion, while *ApoE* loss leads to ECM remodeling with fibrotic properties in mouse aorta [17]. Thus, an general *ApoE* knock out (*ApoE*−/−) mouse model, which is a canonical mouse model for researches on atherosclerosis, hypercholesterolemia, hyperlipidemia and lipid metabolism, will also allow the study of the clinical relevance of ECM reprogramming during tumor progression. Therefore, we used *ApoE*−/− mice to explore whether *ApoE* loss was involved in ECM alterations and if these alterations drive ovarian cancer progression. Our results indicated that the ECM in the abdominal cavity of ApoE−/− mice displayed a remodeled phenotype, and this altered microenvironment promoted the malignant progression of ovarian cancer.

## Methods

### Antibodies and reagents

The following antibodies were used: Col1a2 (14695), FN1 (15613) and GAPDH (10494) from ProteinTech (USA); MMP-9 (AF909, R&D); MMP-10 (NB100–92182, Novus); p-FAK (Y397, ab39967), LOX (ab174316) and Paxillin (ab32084) from Abcam; p-Erk1/2 (Thr202/Tyr204, 4377), p-Src (Y416, 2101), AlexaFluor goat anti-rabbit IgG (594 conjugate, 8889) and sheep anti-rabbit HRP-linked IgG (7074) from Cell Signaling. Reagents included β-aminopropionitrile (BAPN), PKH26 Red Fluorescent Cell Linker Mini Kit (Sigma), PD-325901 (MedChem Express), Hydroxyproline Detection Kit (Nanjing Jiancheng Bioengineering Institute), Rat tail collagen (Invitrogen), Cell counting Kit-8 (Dojindo) and Matrigel matrix (BD).

### Mice studies

Wild Type (WT) (C57BL6) and *ApoE*−/− (B6.129-Apoe^tm1Smoc^) female mice were purchased from Beijing HFK Bioscience. Mice were housed and maintained under specific pathogen-free conditions. For establishment of allografts, ID8-Luciferase cells (1*10^7^) were labeled using PKH26 Cell Linker, and resuspended in 100 μl PBS before intraperitoneal injection into 20-week-old WT and *ApoE*−/− mice. For BAPN treatment (30 mg/kg, i.p.), *ApoE−/−* mice were treated every day for one month. Two weeks after the last day of BAPN treatment, ID8-Luciferase cells (1*10^7^) were intraperitoneally injected. For PD-0325901 treatment, following tumor establishment, *ApoE−/−* mice were randomly assigned the second day and were treated with PD0325901 (25 mg/kg, p.o.) or PBS as a control every day for 2 weeks. The tumors were measured twice a week by quantification of luciferase expression and the animals were sacrificed at the indicated time.

### Cell manipulations

ID8 cells were a kind gift from the The University of Kansas Medical Center, and SKOV3 and OV-90 cells were obtained from the American Type Culture Collection. DMEM, Fetal Bovine Serum (FBS), Insulin-Transferrin-Selenium (ITS), Mycoy’5A medium were purchased from Invitrogen. MCDB105 and 199 medium were purchased from Sigma. ID8 cells were maintained in DMEM supplemented with 4% FBS and 1*ITS. ID8-Luciferase cells were acquired through transfection of lentivirus overexpressing the luciferase reporter gene into ID8 cells. SKOV3 cells were cultured in McCoy’s 5A medium with 10% FBS. OV-90 cells were cultured in MCDB105/199 medium with 10% FBS.

### Real-time quantitative PCR (qPCR)

Reverse transcription of 1 μg of total RNA isolated from the indicated tissues was performed using the PrimeScript™ RT reagent Kit (Takara). The cDNA was subjected to qPCR using an iTAQ™ Universal SYBR^®^ Green Supermix (BIO-RAD). The primer sets used were as follows: Col1a1 (Forward, GCTCCTCTTAGGGGCCACT; Reverse, CCACGTCTCACCATTGGGG), Col1a2 (Forward, GTAACTTCGTGCCTAGCAACA; Reverse, CCTTTGTCAGAATACTGAGCAGC), FN (Forward, ATGTGGACCCCTCCTGATAGT; Reverse, GCCCAGTGATTTCAGCAAAGG), LOX (Forward, TCTTCTGCTGCGTGACAACC; Reverse, GAGAAACCAGCTTGGAACCAG). The primer set for 18S rRNA (Forward, AGGGGAGAGCGGGTAAGAGA; Reverse, GGACAGGACTAGGCGGAACA) was used as a control. Real-time qPCR results were calculated using the delta-delta-Ct (ddCt) algorithm.

### Collagen staining

Hydroxyproline was extracted and quantified in 20–30 mg of tissue (wet weight) or 300 μl of serum as previously described [18]. Paired primary and metastatic lesions from nine patients with grade III/IV high-grade serous ovarian cancer were included. The histology and grade were confirmed by two licensed pathologists. Fresh human and mouse tissues were fixed, paraffin-imbedded and prepared for sections. For picrosirius red analysis, sections were stained with 0.1% picrosirius red and counterstained with Weigert’s hematoxylin. Masson Trichrome was performed according to the manufacturer’s instruction (Sigma). Serial images were analyzed and quantified by the software Image-pro plus 6.0 (Media Cybernetics, Inc., USA). Three fields were randomly selected and the percentages of positive-stained area were calculated.

### Adhesion assay

For in vivo adhesion assays, ID8 cells were labeled with PKH26 red fluorescent Cell Linker, and a single-cell suspension (5*10^6^ in 0.2 ml PBS) was intraperitoneally injected. After four hours, the omentum was excised after sacrifice. Tissues were rinsed three times with PBS to remove non-adhered cells, and images were captured using a fluorescence microscopy (Olympus DP73, Tokyo, Japan). Then, the omentum was digested in 5% NP-40 for 30 min at 37 °C. After scraping with a spatula, all removed cells were collected by centrifugation and the total fluorescence intensity was quantified. For the in vitro adhesion assay, SKOV3 and OV-90 cells were trypsinized and then suspended in cultured medium containing 50 mM Hepes and 1 mg/ml BSA at a concentration of 5*10^5^ cells/ml, from which 0.1 ml was added to each well of 6-well plate. The cells were allowed to adhere for 40 min. Subsequently, the unbound cells were removed by washing with PBS gently. Cells were fixed in 10% formalin and stained with 0.1% crystal violet. Dye was extracted with 10% acetic acid and the relative adhesion capacity was determined by reading at 595 nm [19].

### Immunofluorescence staining, immunohistochemistry (IHC) and western blot analysis

For immunofluorescence staining, the tumor lesions were snap-frozen with OCT (Sakura Fineteck), and prepared as 7 μM cryo-sections. The sections were fixed with ice acetone and SKOV3/OV-90 cells were fixed with 4% paraformaldehyde. Following permeabilization with 0.1% Triton X-100 (Sigma), fixed sections and cells were blocked with 5% BSA in PBS and incubated with the indicated primary antibodies. Sections and cells were then incubated with AlexaFluor goat anti-rabbit IgG (594 conjugate) and nuclei were stained with DAPI (Invitrogen). Images were captured by fluorescence microscopy (Olympus, Japan). For IHC, paraffin sections were deparaffinized and rehydrated, followed by antigen retrieval and incubation with primary antibodies. HRP-based detection reagents were used, and the immunostaining intensity was scored as previously described [20]. Homogenized tissues and cells were lysed in RIPA buffer with protease inhibitors (Roche) and the protein preparations were subjected to western blot analysis as described previously.

### Wound healing assay and Matrigel invasion assay

SKOV3 and OV-90 cells were seeded on dishes with or without Collagen pre-coating. For the wound healing assay, the cellular layer was scratched using a plastic pipette tip. The migration of the cells at the edge of the scratch was analyzed after the indicated time. For the Matrigel invasion assay, following 36 h of culture and 12 h of serum-starved treatment, 1*10^4^ cells were plated into a Matrigel (dilution, 1:7) pre-coated trans-well insert (Corning) in serum-free DMEM, and the bottom chamber contained DMEM with 10% FBS. Cells were allowed to invade through the Matrigel-coated inserts for 36 h. The wound closure efficiency and the number of cells that had invaded were quantified using ImageJ software.

### Cell proliferation assay

Cells (5000) were seeded in triplicates on 96-well plates with or without collagen pre-coating, and those cultured on pre-coated plates were treated with DMSO or PD-325901 (50 nM) for the indicated time. After treatment, cell numbers were quantified using the Cell Counting Kit-8 assay.

### Cytokine/chemokine array

The supernatant of the ascites was collected, and snap-frozen in liquid nitrogen and stored at − 20 °C until analysis. Cytokines and chemokines were measured using the Mouse Cytokine Antibody Array (RayBiotech, QAM-CAA-4000), which includes 200 secreted proteins. The data were analyzed using the Quantibody® Q-Analyzer software, and the pathways were studied using the KEGG database.

### Data analysis

Student’s *t* tests and Mann-Whitney tests were used to compare the statistical significance between two groups. One-way ANOVA (Dunnett post-test) was used for multiple comparison analysis. *P* < 0.05 was considered statistically significant. Values are expressed as the means and standard deviation unless otherwise stated. Graphs and analyses were performed using the GraphPad Prism software.

## Results

### ECM accumulation is associated with tumorigenesis and progression in human ovarian cancer

**Fig. 1:**
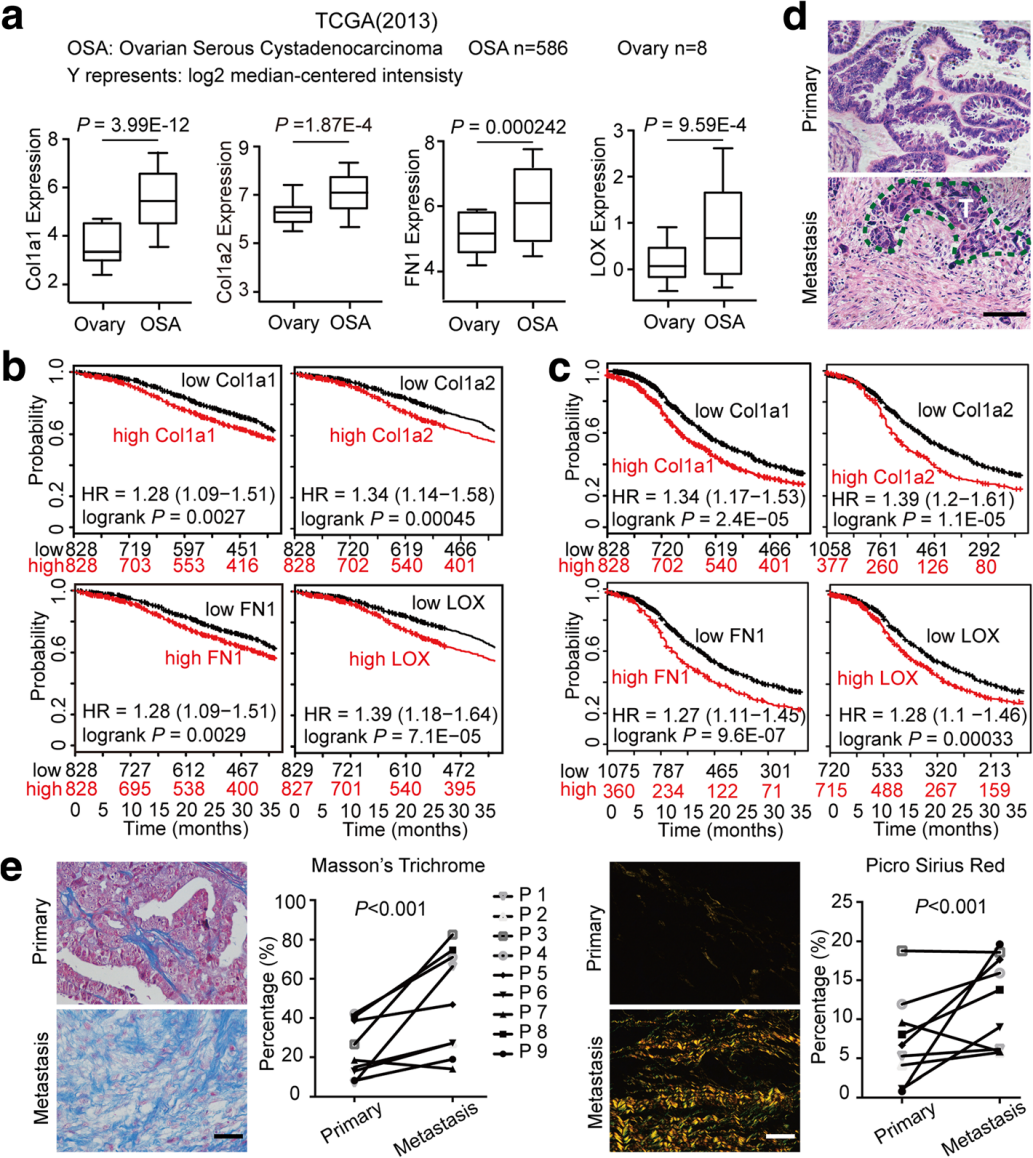
ECM alteration is associated with the tumorigenesis and progression of human ovarian cancer. (**a**) Col1a1, Col1a2, FN1 and LOX transcript expression levels in normal ovary and ovarian cancer tissues in published TCGA data (Oncomine). Center line represents median values, box limits are the 25th and 75th percentiles, and whiskers represent the minimum and maximum values. (**b**, **c**) Kaplan-Meier analyses of the 3-year OS (**b**) and PFS (**c**) in ovarian cancer patients according to Col1a1, Col1a2, FN1 and LOX expression levels. These data were dichotomized at the median value into high and low expressing groups. (**d**) Representative images of H&E staining exhibit the histology of paired primary and metastatic lesions from ovarian cancer patients. The dashed line (green) delimits the tumor tissues. T represents tumor. (**e**) Representative images and positive-stained percentage of Masson’s Trichrome (left) and Picrosirius Red (right) staining of primary lesions and metastases from ovarian cancer patients. Bar represents 50 μm

To explore the ECM alterations during tumorigenesis, we used the Oncomine database (https://www.oncomine.org) to analyze published transcriptional data and found that the transcriptional levels of several ECM components, including collagen 1a1 (Col1a1), Col1a2, fibronectin 1 (FN1) and lysyl oxidase (LOX), were robustly increased in ovarian cancer compared to normal ovaries (Fig. 1, Additional file 1: Figure S1A). Also, the alterations of these genes showed clinical relevance. High mRNA expression of Col1a1, Col1a2, FN1 and LOX was linked to a poor prognosis in ovarian cancer, as shown by the reduced overall survival (OS) and progression free survival (PFS) (http://kmplot.com) (Fig. 1b, c). The improved OS and PFS in low-expressers implied a delay in recurrence. To investigate the ECM during ovarian cancer progression, we examined the histology of paired primary lesions and metastases from ovarian cancer patients. Indeed, compared to primary lesions, metastatic tumors showed a desmoplastic stromal response, which is characterized by dense ECM around tumor lesions (Fig. 1d). As collagen is the main structural protein in the extracellular space, we next investigated whether alterations of collagen composition occurred during tumor progression. By Masson’s Trichrome and Picro Sirius Red staining, we found a remarkable increase in fibrillar collagen in the metastatic tissues compared to primary tumors (Fig. 1e). Overall, these results indicate that ECM alterations, especially collagen deposition, frequently occurs during the malignant progression of ovarian cancer.

### *ApoE* loss leads to altered peritoneal ECM composition

**Fig. 2:**
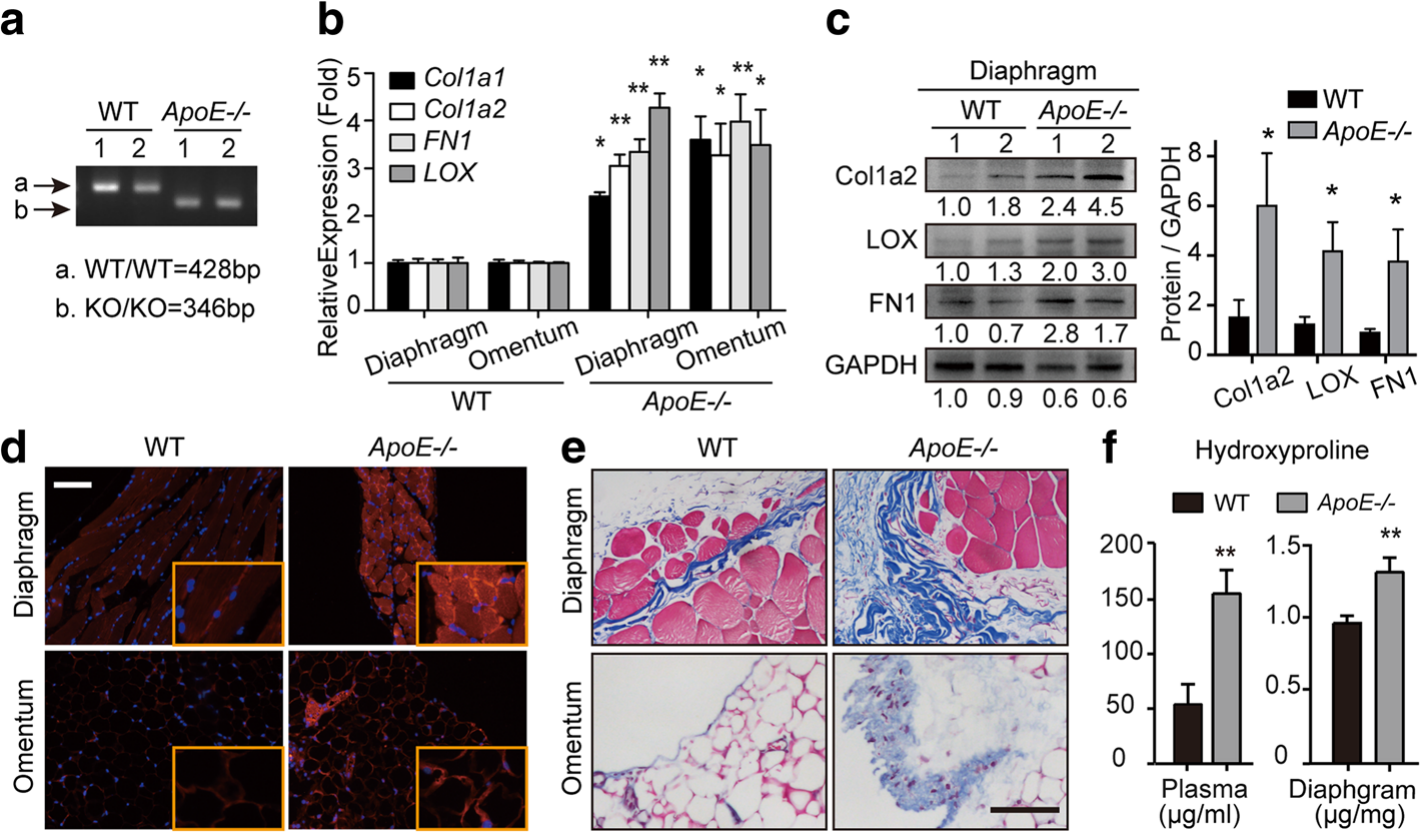
*ApoE* loss leads to altered peritoneal ECM composition. (**a**) Agarose gel electrophoresis of *ApoE* gene. (**b**) The mRNA expression of Collagen1, FN1 and LOX in the excised diaphragm and omentum of WT and *ApoE*−/− mice. (**c**) Western blot analysis of Col1a2, LOX and FN1 protein levels in the diaphragm. 1 and 2 represent different samples. (**d**) Frozen sections were immunoassayed for LOX (red) and nuclei were stained with Hoechst (blue). (**e**) The representative images of Masson’s Trichrome staining in the diaphragm and omentum from WT and *ApoE*−/− mice. (**f**) The plasma and diaphragm were analyzed for hydroxyproline content. Error bars represent the SD of triplicate experiments. Bar represents 50 μm. *P < 0.05; **P < 0.005

According to previous reports, the mRNA expression of *ApoE* showed a moderate increase during ovarian cancer tumorigenesis (Additional file 1: Figure S1B). To investigate whether *ApoE* loss mediates intraperitoneal ECM alterations, we measured the ECM components of the diaphragm and omentum, which are where ovarian cancer likely spreads (Fig. 2a). qPCR showed an ~ 2-fold increase in the expression of Col1a1, Col1a2, FN1 and LOX in *ApoE−/−* mice compared to WT controls (Fig. 2b). Consistently, western blot analysis indicated enhanced protein levels of Col1a2, LOX and FN1 in the diaphragm of *ApoE−/−* mice (Fig. 2c). The protein induction of LOX, an amine oxidase responsible for crosslinking and stabilization of adjacent collagens, was further confirmed by immunofluorescence analysis (Fig. 2d). We next explored the expression levels and structure of collagens using Masson’s Trichrome stain. The representative sections exhibited a remarkable increase in fibrous collagen (blue stain) in *ApoE−/−* mice compared to WT (Fig. 2e). Furthermore, hydroxyproline, a surrogate for collagen, was increased in both the plasma (157.4 ± 26.2 vs 59.5 ± 21.9 μg/ml; mean ± SD, *n* = 5) and diaphragm (1.29 ± 0.12 vs 0.94 ± 0.06 μg/mg; mean ± SD, n = 5) of *ApoE*−/− mice compared to WT mice (Fig. 2f). Taken together, *ApoE−/−* mice displays ECM accumulation, especially collagen deposition, in the peritoneal cavity.

### The altered peritoneal microenvironment accelerates ovarian cancer progression

**Fig. 3:**
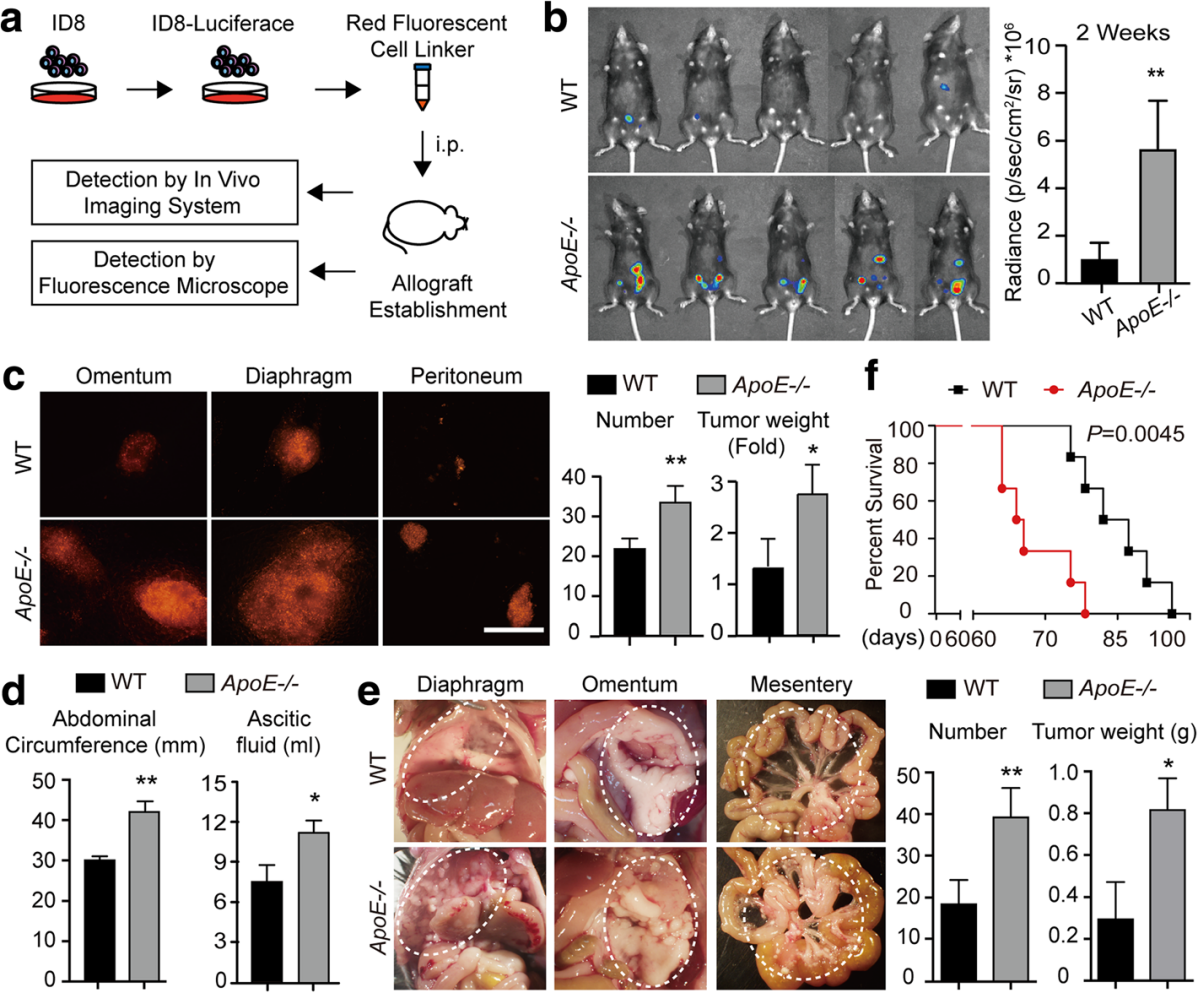
The remodeled peritoneal microenvironment accelerates ovarian cancer progression. (**a**) Flowchart of the in vivo experiments. (**b**) Luciferase bioluminescence was determined in WT and *ApoE*−/− mice two weeks after ID8 allografts engraftment. Graph represents the mean radiance for each group (*n* = 5). (**c**) The omentum, peritoneum and diaphragm of WT and *ApoE*−/− mice were excised and analyzed under a fluorescence microscope (left). The number and weight of the tumor lesions were measured (right). Bar represents 200 μm. (**d**) The tumor burden was assayed by the abdominal circumference and ascetic fluid content. (**e**) Representative images (left) and quantification (right) of tumor lesions dispersed in the diaphragm, omentum and mesentery. Dashed circles delimit the tumor tissues. (**f**) Survival rate of WT and *ApoE*−/− mice with ID8 allografts. Error bars represent the SD of the experimental data from five mice. *P < 0.05; **P < 0.005

To explore whether the altered ECM in the peritoneal microenvironment of *ApoE*−/− mice could modulate the progression of ovarian cancer, we used an ID8 intraperitoneal allograft model. ID8 is a spontaneously tumorigenic mouse ovarian surface epithelial cell line that is not rejected by the host immune system. To monitor tumor progression, ID8 cells were stably transfected with a luciferase reporter, and the cellular membrane was labeled with PKH26 Cell Tracking Dye (Fig. 3a). Two weeks after tumor engraftment, in vivo bioluminescent imaging showed increased luciferase expression in *ApoE−/−* mice compared to WT (mean radiance: 5.67*10^6^ vs 0.92*10^6^ p/s/cm^2^/sr, *P* < 0.005) (Fig. 3b). Then a cohort of mice was sacrificed and dissected under a fluorescent microscope. Likewise, the peritoneal cavity of *ApoE*−/− mice displayed ~ 2-fold and 2.64-fold increases in tumor number and weight, respectively (Fig. 3c). In the advanced stage of malignancy, morbid *ApoE−/−* mice presented obvious abdominal distension, caused by excessive hemorrhagic ascites, with a 42 mm abdominal circumference and an 11.5 ml ascites volume on average, compared to 30 mm (*P* < 0.005) and 7.5 ml (*P* < 0.05) in WT mice (Fig. 3d). By anatomy and quantification, morbid *ApoE−/−* mice displayed a heavier tumor burden compared to WT control (Fig. 3e). Supporting those observations, *ApoE*−/− mice had a shortened survival compared to WT mice (Fig. 3f). Together these results revealed that *ApoE* loss accelerated the malignant progression of ID8 intraperitoneal allografts.

### ECM accumulation enhances the invasive behaviors of ovarian cancer cells

**Fig. 4:**
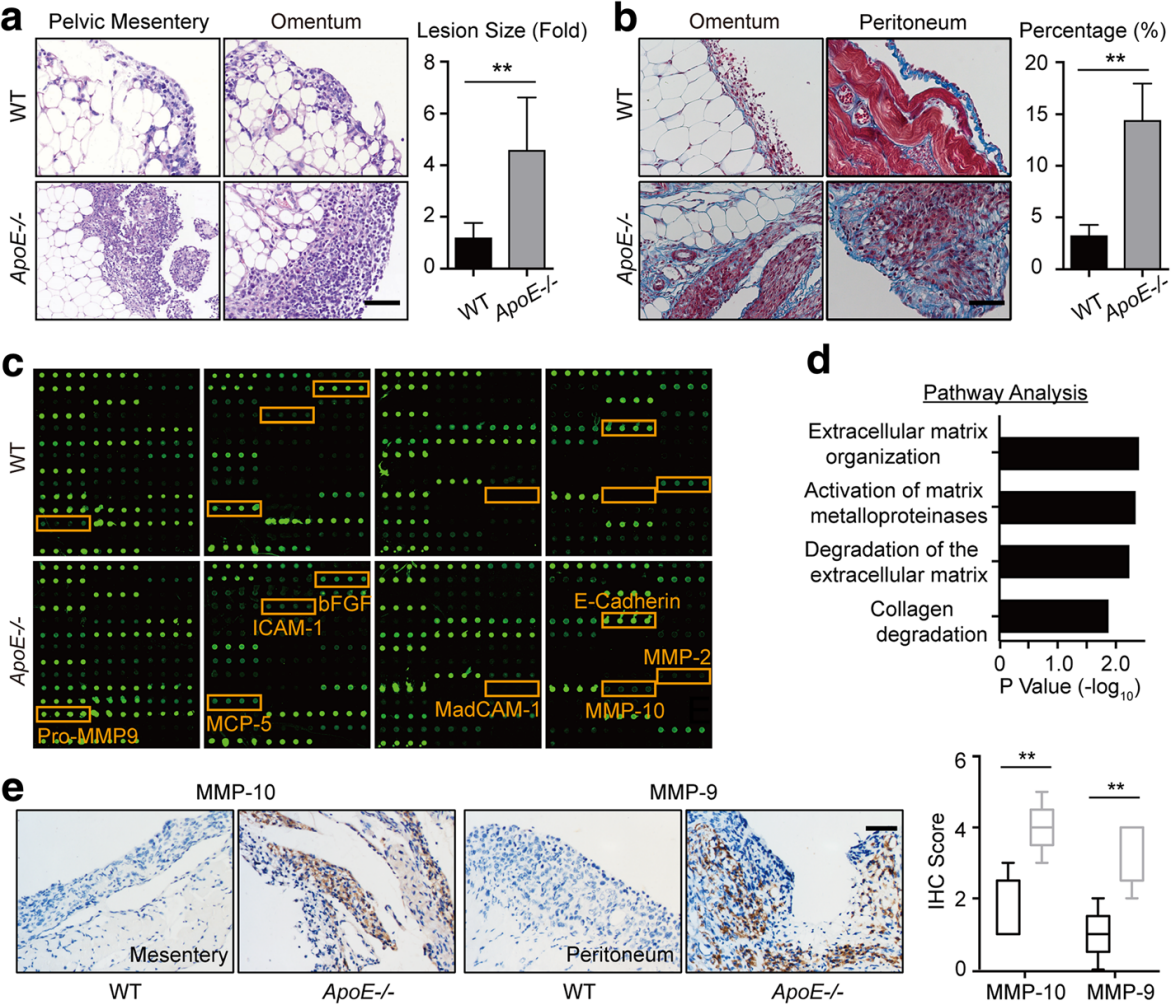
Remodeled ECM enhances the invasive behaviors of ovarian cancer cells. (**a**) H&E stain of the tumor lesions from WT and *ApoE*−/− mice at two weeks post ID8 engraftment (left). Graph represents the mean size of the lesions calculated from ten random fields (right). (**b**) Masson’s Trichrome stain of tumor lesions (left). The mean percentage of positive regions in ten random fields was calculated (right). (**c**) The cytokines/chemokines profile in the supernatants of ascites from WT and *ApoE*−/− mice. Four groups of mouse cytokine dot-blot arrays are shown. Dot-blots with significant changes are shown in boxed areas (red). (**d**) The top four pathways enriched among the molecules with significant changes using KEGG pathway analysis. (**e**) MMP-10 and MMP-9 protein expression in tumor lesions, determined by blinded IHC analysis (left). Box plot of the IHC score of MMP-10 and MMP-9 (right). Box represents the 25th–75th percentile while whiskers indicate the 5th–95th percentile. The black box represents tumor lesions from WT mice and the grey box represents tumor lesions from *ApoE*−/− mice. Each experiment included data from 5 mice. Bar represents 50 μm. *P < 0.05; **P < 0.005

To assess the mechanism by which ECM alterations promote ovarian cancer progression, we further evaluated the tumor specimens from *ApoE*−/− and WT mice. Hematoxylin and eosin (H&E) staining showed that the sizes of tumor lesion at two weeks were significantly larger in *ApoE*−/− mice (Fig. 4a). Also, lesions in the *ApoE*−/− mice exhibited increased collagenous tissues, especially around the tumor lesions, indicated by Masson’s Trichrome stain (Fig. 4b). Therefore, we reasoned that the altered ECM might stimulate tumor progression by providing a favorable niche for cancer cells. Because ascites facilitate peritoneal spreading of ovarian cancer [2], we then analyzed the cytokines/chemokines in the ascites using mouse-specific cytokine profiling arrays. Though there might be cellular contamination of the secretome due to the increase of E-cadherin, a group of molecules that potentiate ECM reorganization and MMP activation stood out (Fig. 4c, d and Additional file 2: Table S1). Since MMPs are important regulators of cell invasion, we measured MMP-10 and MMP-9 expressions using IHC. Our results confirmed the markedly elevated expression of MMP-10 and MMP-9 in the tumor lesions of *ApoE*−/− mice compared to that in WT mice (Fig. 4e). Although ApoE could suppress angiogenesis in melanoma [13], we did not observe significant differences of angiogenesis in tumor lesions from *ApoE−/−* and WT mice (Additional file 3: Figure S2). In vitro, collagen pre-coating of substrates could stimulate the proliferation of SKOV3 and OV-90 cells, two human ovarian cancer cell lines (Additional file 4: Figure S3A). The protein expressions of MMP-10 and MMP-9 were increased in cells cultured on collagen pre-coated substrates (Additional file 4: Figure S3B), and these cells exhibited enhanced invasive potentials (Additional file 4: Figure S3C-F). Overall, ECM accumulation, especially collagen accumulation, enhances the invasive potentials of ovarian cancer cells.

### BAPN treatment delays ovarian cancer progression by reducing adhesions

**Fig. 5:**
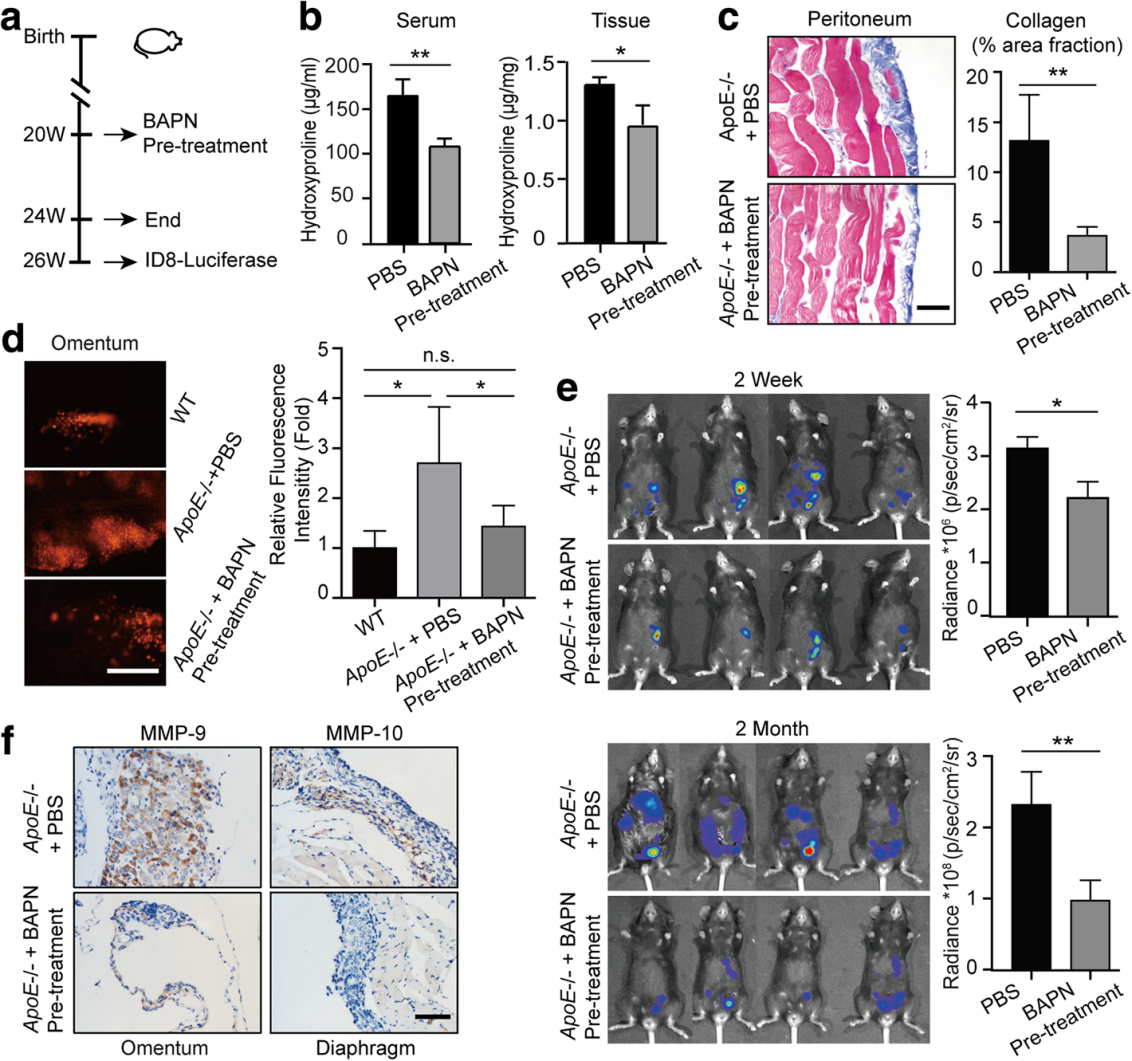
BAPN treatment delays ovarian cancer progression by reducing adhesions. (**a**) Experimental design: PBS or BAPN was intraperitoneally administrated to 20-weeks-old female *ApoE*−/− mice each day and continued for four weeks. A cohort of mice was sacrificed for further experiments. For the remaining mice, the drug treatment was stopped for two weeks before the establishment of ID8 allografts. (**b**) Hydroxyproline was measured in the plasma and diaphragm. (**c**) Masson’s Trichrome stain after BAPN treatment (left). The positive-staining percentage of 10 random fields was calculated (right). Bar represents 50 μm. (**d**) Cells adhesive to the omentum were analyzed four hours after ID8 intraperitoneal injection by fluorescence microscopy (left). The adhesive cells were determined from the total fluorescent intensity after digestion (right). Bar represents 200 μm. (**e**) In vivo luciferase measured at two weeks (top) and two months (bottom) post establishment in *ApoE*−/− mice with PBS or BAPN pre-treatment. Quantification of luminescence is represented as the radiance. (**f**) MMP-9 expression measured by IHC in tumor lesions of *ApoE*−/− mice with PBS or BAPN treatment. Each experiment includes data from 4 mice. Bar represents 50 μm. *P < 0.05; **P < 0.005

LOX is a key regulator of collagen homeostasis and fibronectin expression [21]. β-Aminopropionitrile (BAPN), a specific inhibitor of LOX activity, can ameliorate fibrosis by suppressing collagen synthesis [22] and reducing FN expression [23]. Therefore, we used BAPN to decrease the synthesis and secretion of ECM for reversing ECM reprogramming in *ApoE−/−* mice. BAPN treatment was initiated in 20-week-old *ApoE−/−* mice and sustained for one month prior to tumor establishment (Fig. 5a). To evaluate the efficiency of BAPN treatment, a group of *ApoE−/−* mice was sacrificed and compared to PBS-treated mice (CTRL). Hydroxyproline was measured and indicated that BAPN treatment curtailed the collagen content in the serum (106 ± 9.4 vs 168 ± 17.9 μg/ml; mean ± SD, *n* = 5) and diaphragm (0.91 ± 0.16 vs 1.28 ± 0.06 μg/mg; mean ± SD, n = 5) (Fig. 5b). Also, Masson’s Trichrome stain exhibited decreased collagen deposition (Fig. 5c). ID8 allografts were established two weeks after the last day of BAPN pre-treatment. Since the first step of the metastatic cascade is the adhesion of cancer cells, we sacrificed a cohort of mice four hours after ID8 intraperitoneal injection to assess adhesions in vivo. Whereas more cancer cells were attached to the tissues surfaces in *ApoE*−/− mice compared to WT controls, pre-treatment with BAPN robustly attenuated the adhesions compared to CTRL mice (Fig. 5d). By monitoring tumor progression using in vivo bioluminescence, we found a significant decrease in the tumor burden in the BAPN-treated group compared to the CTRL group, both at two weeks and two months post allografts establishment (2 weeks: 2.11 vs 3.13*10^6^ p/s/cm^2^/sr, *P < 0.05*; 2 months: 0.93 vs 2.32*10^8^ p/s/cm^2^/sr, *P < 0.005*) (Fig. 5e). Furthermore, IHC staining showed significantly diminished expression of MMP-9 and MMP-10 with BAPN treatment (Fig. 5f). Overall, these results suggest that ECM accumulation stimulates ovarian cancer progression by promoting adhesion.

### Adhesion-mediated signaling promotes malignancy of ovarian cancer via FAK-ERK-MMP pathway activation

**Fig. 6:**
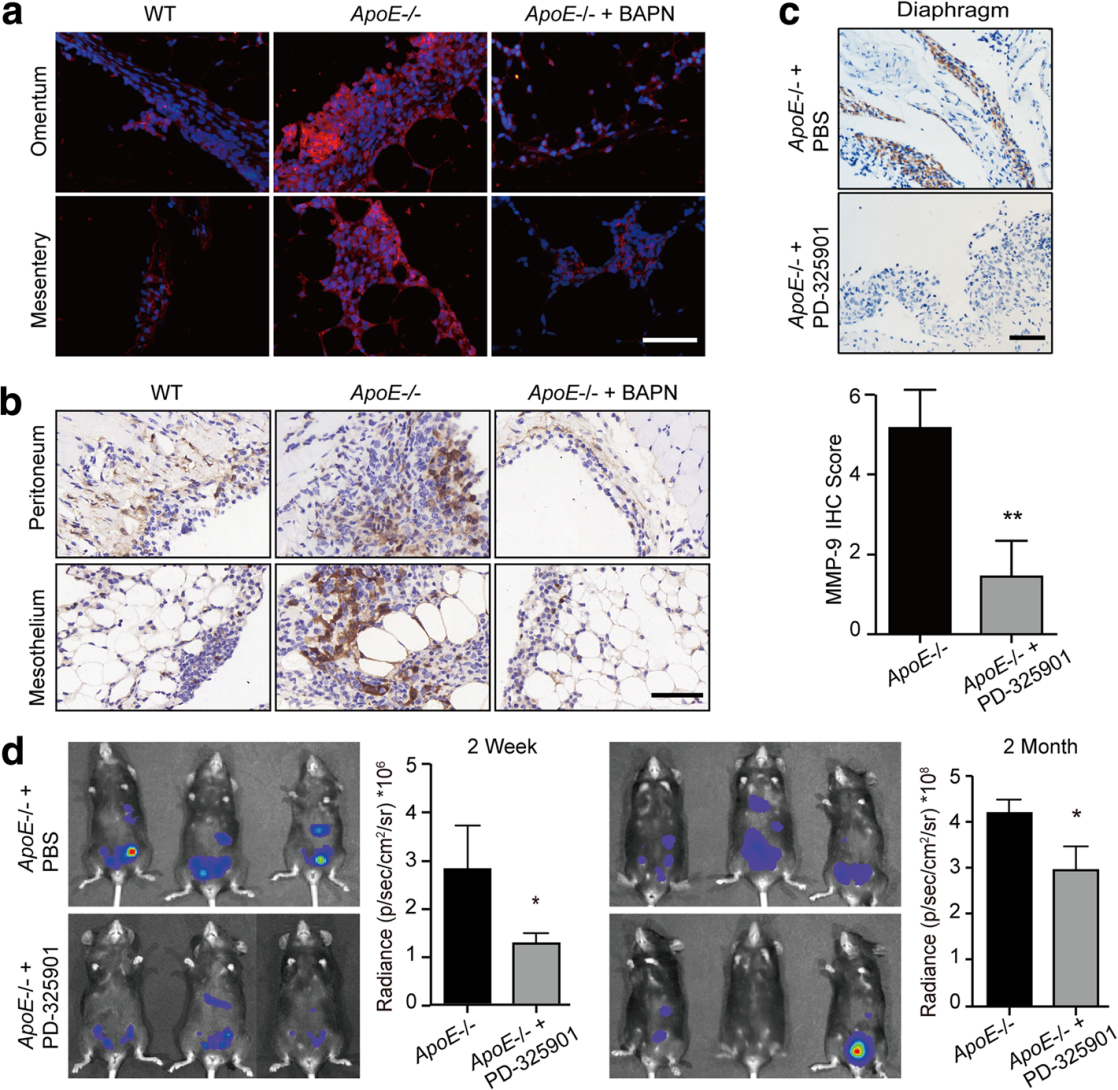
Remodeled ECM promotes malignancy of ovarian cancer via FAK-ERK-MMP activation. (**a**) Specimens from Fig. 5D were immunoassayed for p-FAK^Y397^ (red), and nuclei were stained with Hoechst (blue). (**b**) IHC of active ERK (p-p44/42 MAPK^Thr202/Tyr204^) in tumor lesions from WT and *ApoE*−/− mice with PBS or BAPN treatment two weeks after ID8 engraftment. (**c**) PBS or PD-325901 was administrated to *ApoE*−/− mice once ID8-Luciferase cells were intraperitoneally injected and treatment continued for one month. The representative images (top) and quantification data (bottom) of MMP-9 protein expression in tumor lesions two months after ID8 engraftment. (**d**) In vivo luciferase expression was determined two weeks or two months post treatment. Luminescence (right panel) is represented as the radiance (p/s/cm^2^/sr). Each experiment included data from 4 mice. Bar represents 50 μm. *P < 0.05; **P < 0.005

Increased collagen-matrix density enhances adhesion and activates the FAK-Rho-ERK pathway, which enhances the invasive phenotype of mammary epithelial cells [24]. Focal adhesion kinase (FAK), a mediator of cell adhesion, motility and angiogenesis, can transmit extracellular signals into cells and thus facilitate various neoplastic processes [25]. In our model, immunofluorescence analysis at the early stage of the tumor establishment showed significantly increased FAK activation in the tumor cells from *ApoE*−/− mice compared to WT controls (Fig. 6a). Also, adhesions of SKOV3 and OV-90 cells were dramatically enhanced with collagen pre-coated substrates, and immunofluorescence analysis confirmed the collagen signaling-induced Paxillin-positive adhesions (Additional file 5: Figure S4A, B). Notably, phospho-FAK was significantly increased, and its signaling partner, Src, was also activated (Additional file 5: Figure S4B, C). To explore the FAK-mediated signaling underpinning the malignant phenotype of ovarian cancer cells, we measured the activity of ERK in tumor lesions with IHC staining. Compared to WT mice, *ApoE−/−* mice showed active ERK signaling in tumor lesions (Fig. 6b). Consistently, SKOV3 and OV-90 cells seeded on collagen pre-coated substrates also exhibited ERK activation (Additional file 5: Figure S4B, C). To explore the FAK-ERK activation in ovarian cancer progression, we treated *ApoE−/−* mice after ID8 engraftment with the MEK inhibitor (MEKi) PD-325901 to selectively inhibit ERK phosphorylation. The treatment continued for one month. By IHC staining, we found that PD-325901 robustly diminished MMP-9 protein expression in the tumor lesions (Fig. 6c). Also, PD-325901 significantly curtailed the collagen signaling-induced cell proliferation, and decreased the synthesis of MMP-9 and MMP-10 (Additional file 5: Figure S4D, E). These results were consistent with previous reports showing that MMPs are regulated by the MAPK/ERK pathway [26, 27]. Consistently, BAPN pre-treatment in *ApoE*−/− mice caused decreased FAK activation accompanied by diminished cancer cells adhesions (Fig. 6a). Also, BAPN-treated mice developed smaller metastases with weak p-ERK expression (Fig. 6b). More importantly, our results demonstrated that PD-325901 treatment significantly delayed the tumor progression in *ApoE*−/− mice compared to PBS-treated mice (Fig. 6d). Overall, our results strongly suggest that the remodeled microenvironment in *ApoE*−/− mice enhances malignancy of ovarian cancer by increasing adhesion-mediated FAK-ERK-MMP signaling.

## Discussion

The goal of this study was to assess *ApoE* loss-mediated intraperitoneal ECM reprogramming and the function of the remodeled ECM in ovarian cancer progression. Here we demonstrate that **(1)** *ApoE* knock-out leads to increased expression of several ECM molecules in the peritoneal cavity. **(2)** These molecules collectively remodel the peritoneal microenvironment and stimulate ovarian cancer progression. **(3)** The remodeled ECM promotes the invasive behavior of cancer cells by increasing FAK-ERK-MMP activation. **(4)** ECM reprogramming is associated with tumorigenesis and progression of human ovarian cancer and is negatively correlated with the OS and PFS.

Metastatic ovarian cancer is characterized by extensive seeding within the abdominal cavity. The attachment of ovarian cancer cells to the mesothelial cells that cover the peritoneal cavity is the initial, crucial step of peritoneal implantation. Several events can enhance this process. Oncogenes, such as mutant p53 and ETV5, promote the adhesions of ovarian cancer cells by regulating the expression of adhesion molecules such as integrin β_4_, β_1_ and α_1_ [28, 29]. Also, the binding of CA125 to mesothelin, which is expressed by mesothelial cells, mediates ovarian cancer cell adhesion [30]. An adhesion molecule protein signature can be used as a prognostic tool for ovarian cancer patient outcome [31]. In addition to the surface growth and proximity to the peritoneum of ovarian cancer cells, the peritoneal microenvironment can actually favor the implantation of ovarian cancer cells with dynamic integrin-mediated cell-ECM adhesions [32]. Indeed, ECM alterations were previously linked to disrupted cell polarity, enhanced adhesion, increased cell proliferation, malignant transformation and metastasis [24, 25]. In our study, the analysis of clinical samples showed that ECM accumulation was associated with ovarian cancer tumorigenesis and progression. Also, the altered ECM was significantly correlated with the OS and PFS of ovarian cancer patients. Recent studies demonstrate that ECM reprogramming can not only be induced by the invasive tumor cells, but can also be promoted by stromal cells in the absence of cancer cells [33]. Our research also describes the impact of preceding altered ECM on the malignant progression of ovarian cancer. This remodeled microenvironment promotes adhesions and the invasive behavior of cancer cells. More importantly, clinical evidences showed that chronic pelvic inflammatory disease, endometriosis, aging, obesity and chemotherapy may contribute to peritoneal fibrosis [34–38]. Though further studies will be needed to characterize the ECM alterations induced by these various conditions and investigate their impacts on ovarian cancer progression, it is possible that the ECM alterations induced by systematic treatments actually render a metastatic soil for resident cancer cells, leading to earlier recurrence.

Recently, targeting ECM has been of particular interest as ECM dysregulation emerges as a common driver of tumor progression. Potential therapeutic targets include key ECM proteins or the matrix-remodeling enzymes such LOX and MMPs. In addition to the decrease of mammary tumor growth [25], LOX inhibition has also been reported to reduce cancer cell motility, reverse hypoxia-induced EMT and suppress metastasis [39]. BAPN, as a typical drug to inhibitor LOX, has shown promising anti-cancer effects in preclinical trial [40]. Indeed, BAPN treatment has multifactorial effects, and our results provide more evidence for the protective effects of BAPN. We demonstrated that BAPN treatment decreased the total level of collagen, reduced adhesions and ultimately tempered the invasive potentials of ovarian cancer cells. In recent studies, the well-established role of FAK and ERK activation in the interaction of ovarian cancer cells and ECM fully confirmed the connection of ECM and ovarian cancer malignancy [41, 42]. We described a FAK-ERK-MMP linkage in the early events of metastasis, which drove the malignant progression of ovarian cancer cells. The efficiency of MEKi for suppressing tumor progression provides an impetus for further studies exploring MEKi as a treatment for ovarian cancer patients. *Kras*/*Braf* mutations in low-grade serous ovarian carcinomas (LGSOC) and *MAP3K8* accumulation in high-grade serous ovarian carcinomas (HGSOC) induce constitutive MEK/ERK activation and significantly sensitize ovarian cancer cells to MEKi [43]. Combination of MEKi and traditional chemotherapies also shows synergistic effects on tumor suppression in ovarian cancer [44, 45]. More importantly, new drugs that inhibit MEK are currently undergoing late-stage clinical evaluation [46]. Trametinib, a selective MEKi approved by the US FDA in 2013 and EMA in 2014, is associated with longer PFS and OS in metastatic melanoma patients as monotherapy in a phase 3 clinical trial [47]. Also, MEKi is well tolerated in the treatment of recurrent low-grade serous ovarian or peritoneal carcinoma in a phase 2 study [48], and this exciting finding prompts the ongoing assessment of MEKi in a randomized phase 3 trial. Overall, MEKi treatment exhibits potential benefit in the treatment of ovarian cancers.

However, there are several limitations in our study. The functional loss or mutations in p53 has been associated to the migration and invasion of cancer cells [49, 50]. Almost all HGSOC tumors harbor p53 mutations, whereas, ID8 allografts utilized in our model have wild type p53 and may not perfectly represent the biological process of the disease.

## Conclusions

Our results show that the FAK-ERK activation in cell/matrix adhesion is of broad relevance to the malignant progression of ovarian cancer, and that the efficiency of BAPN or MEKi for tumor suppression indicate the possibility of new anticancer therapeutic combinations that target the intraperitoneal ECM.

## Additional files

Additional file 1: **Figure S1.** ECM is upregulated during tumorigenesis. (TIFF 244 kb)

Additional file 2: **Table S1.** The quantification of secreted factors in the cytokine profiling arrays. (DOC 210 kb)

Additional file 3: **Figure S2.** Angiogenesis does not mediate the malignant progression of ovarian cancer in *ApoE* knock out mice. (TIFF 1354 kb)

Additional file 4: **Figure S3.** Collagen signaling stimulates the malignant phenotype of ovarian cancer cells. (TIFF 3512 kb)

Additional file 5: **Figure S4.** Collagen signaling promotes adhesion and activates FAK-ERK linkage in ovarian cancer cells. (TIFF 1768 kb)

## Abbreviations

ApoE: Apolipoprotein E
BAPN: β-aminopropionitrile
Col: Collagen
ECM: Extracellular matrix
FAK: Focal adhesion kinase
FN: Fibronectin
H&E: Hematoxylin and eosin
IHC: Immunohistochemistry
LOX: Lysyl oxidase
MEKi: MEK inhibitor
OS: Overall survival
PFS: Progress free survival
qPCR: quantitative PCR
WT: Wild type
