# Correction to: FAK-ERK activation in cell/matrix adhesion induced by the loss of apolipoprotein E stimulates the malignant progression of ovarian cancer

**DOI:** 10.1186/s13046-019-1422-6

**Published:** 2019-10-15

**Authors:** Huiling Lai, Xuejiao Zhao, Yu Qin, Yi Ding, Ruqi Chen, Guannan Li, Marilyne Labrie, Zhiyong Ding, Jianfeng Zhou, Junbo Hu, Ding Ma, Yong Fang, Qinglei Gao

**Affiliations:** 1 Cancer Biology Research Center (Key laboratory of the ministry of education), Tongji Hospital, Tongji Medical College, Huazhong University of Science and Technology, Wuhan, 430030 People’s Republic of China; 2 Department of Systems Biology, University of Texas MD Anderson Cancer Center, Houston, TX 77030 USA

**Correction to: J Exp Clin Cancer Res**

**https://doi.org/10.1186/s13046-018-0696-4**

In the original publication of this manuscript [1], Fig. 5E lower panel was incorrect due to an error in the preparation of these figures for publication. It was noticed that in the lower panel of Fig. 5E, one mouse image of ApoE−/− + PBS group (upper) was a photograph coming from ApoE−/− + BAPN pre-treatment group (lower). The corrected figure appears below. We apologize for any confusion this may have caused.

**Fig. 5:**
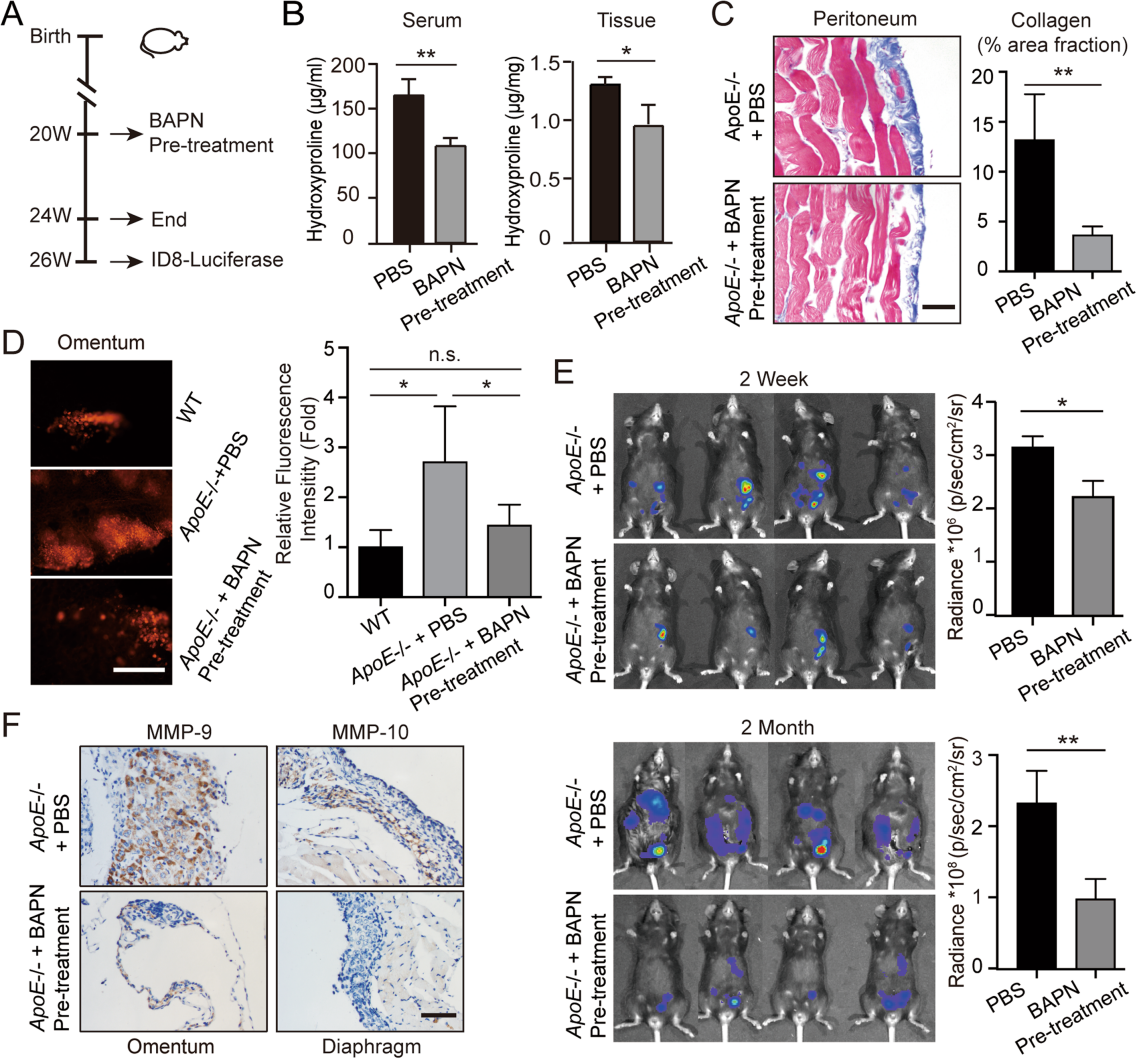
BAPN treatment delays ovarian cancer progression by reducing adhesions. (**a**) Experimental design: PBS or BAPN was intraperitoneally administrated to 20-weeks-old female *ApoE*−/− mice each day and continued for four weeks. A cohort of mice was sacrificed for further experiments. For the remaining mice, the drug treatment was stopped for two weeks before the establishment of ID8 allografts. (**b**) Hydroxyproline was measured in the plasma and diaphragm. (**c**) Masson’s Trichrome stain after BAPN treatment (left). The positive-staining percentage of 10 random fields was calculated (right). Bar represents 50 μm. (**d**) Cells adhesive to the omentum were analyzed four hours after ID8 intraperitoneal injection by fluorescence microscopy (left). The adhesive cells were determined from the total fluorescent intensity after digestion (right). Bar represents 200 μm. (**e**) In vivo luciferase measured at two weeks (top) and two months (bottom) post establishment in *ApoE*−/− mice with PBS or BAPN pre-treatment. Quantification of luminescence is represented as the radiance. (**f**) MMP-9 expression measured by IHC in tumor lesions of *ApoE*−/− mice with PBS or BAPN treatment. Each experiment includes data from 4 mice. Bar represents 50 μm. *P < 0.05; **P < 0.005
