# Supplementary figures and images for: FAK-ERK activation in cell/matrix adhesion induced by the loss of apolipoprotein E stimulates the malignant progression of ovarian cancer
Source: J Exp Clin Cancer Res. 2018 Feb 20;37:32. doi: 10.1186/s13046-018-0696-4 (PMC5819228; doi:10.1186/s13046-018-0696-4)

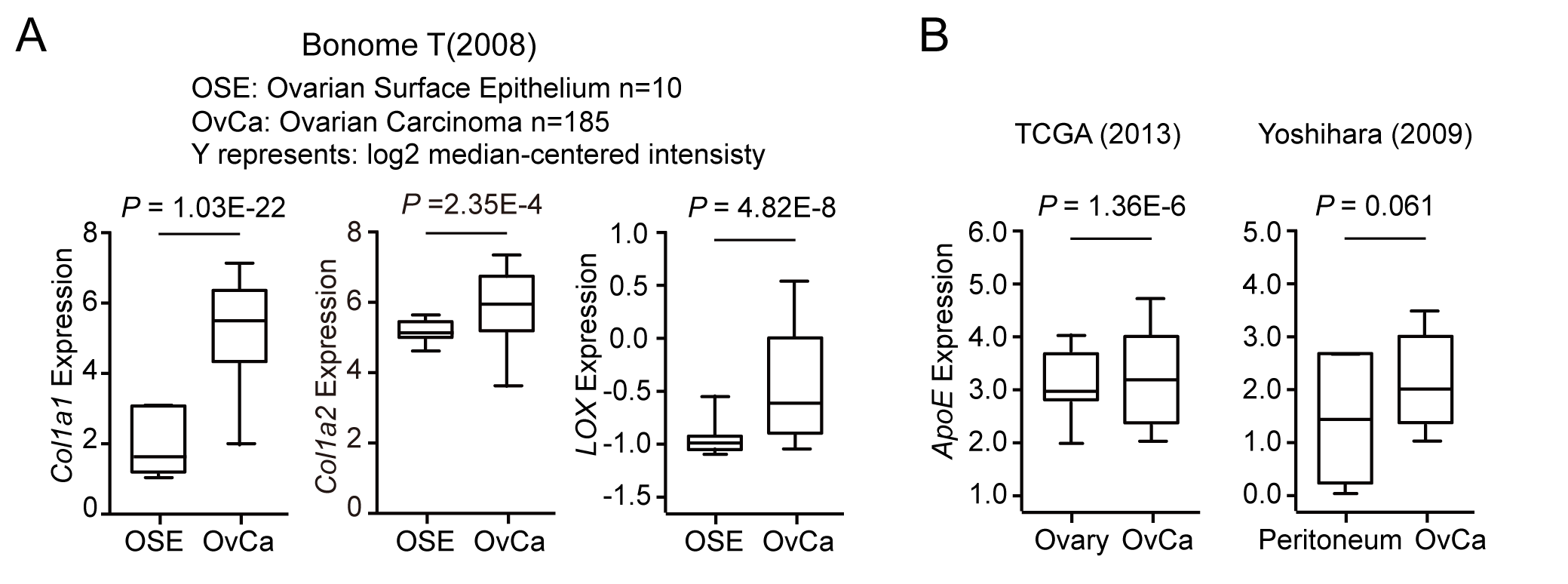

Supplement: Supplementary file 1 — Figure S1. ECM is upregulated during tumorigenesis. (TIFF 244 kb) [file 13046_2018_696_MOESM1_ESM.tif]

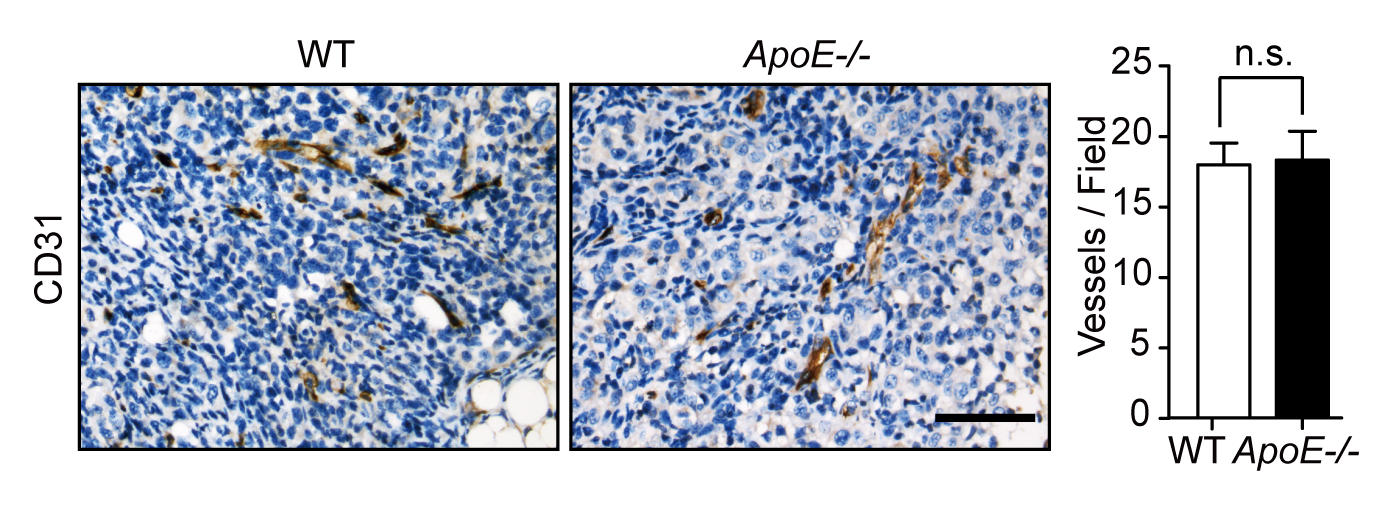

Supplement: Supplementary file 3 — Figure S2. Angiogenesis does not mediate the malignant progression of ovarian cancer in ApoE knock out mice. (TIFF 1354 kb) [file 13046_2018_696_MOESM3_ESM.tif]

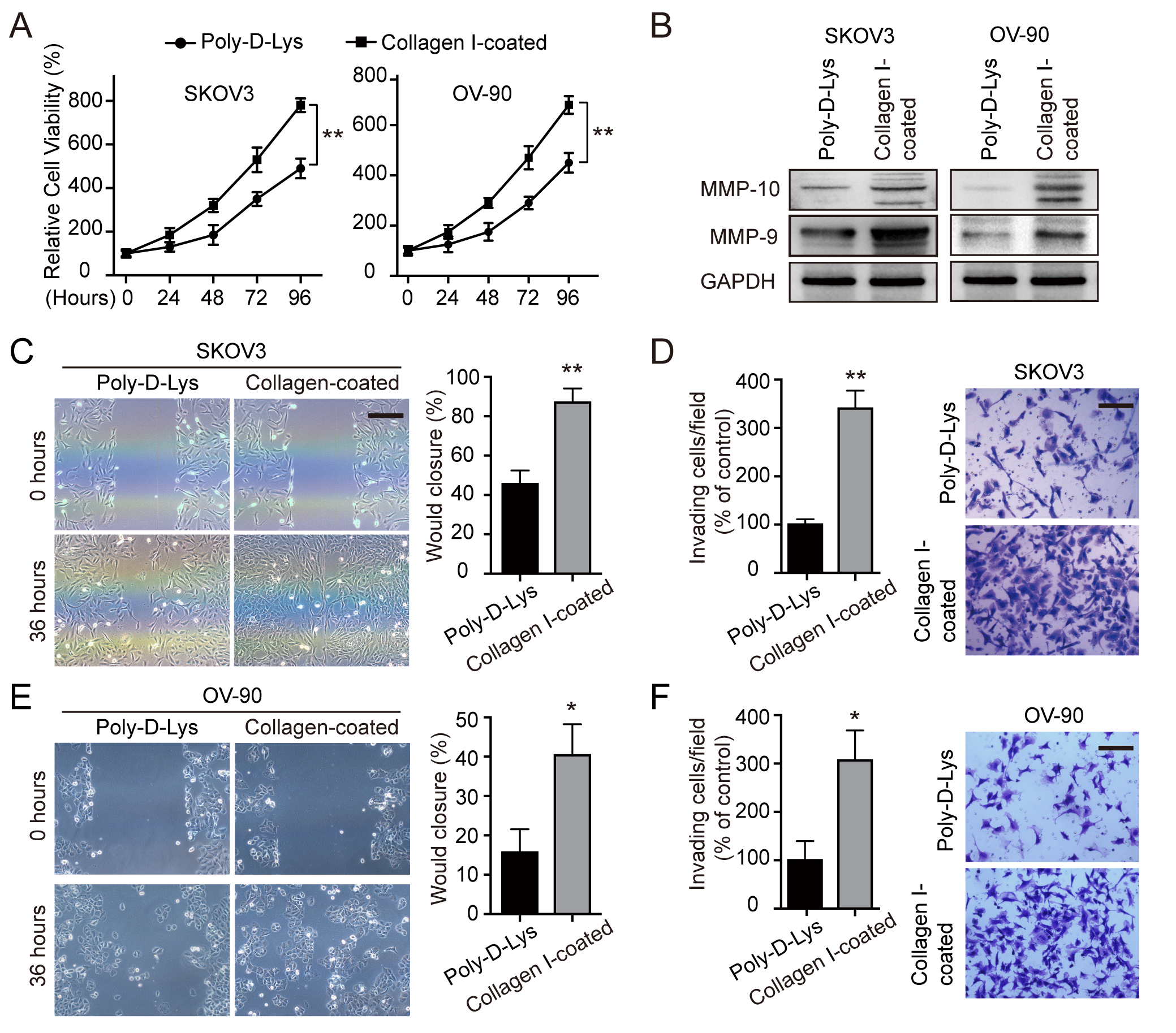

Supplement: Supplementary file 4 — Figure S3. Collagen signaling stimulates the malignant phenotype of ovarian cancer cells. (TIFF 3512 kb) [file 13046_2018_696_MOESM4_ESM.tif]

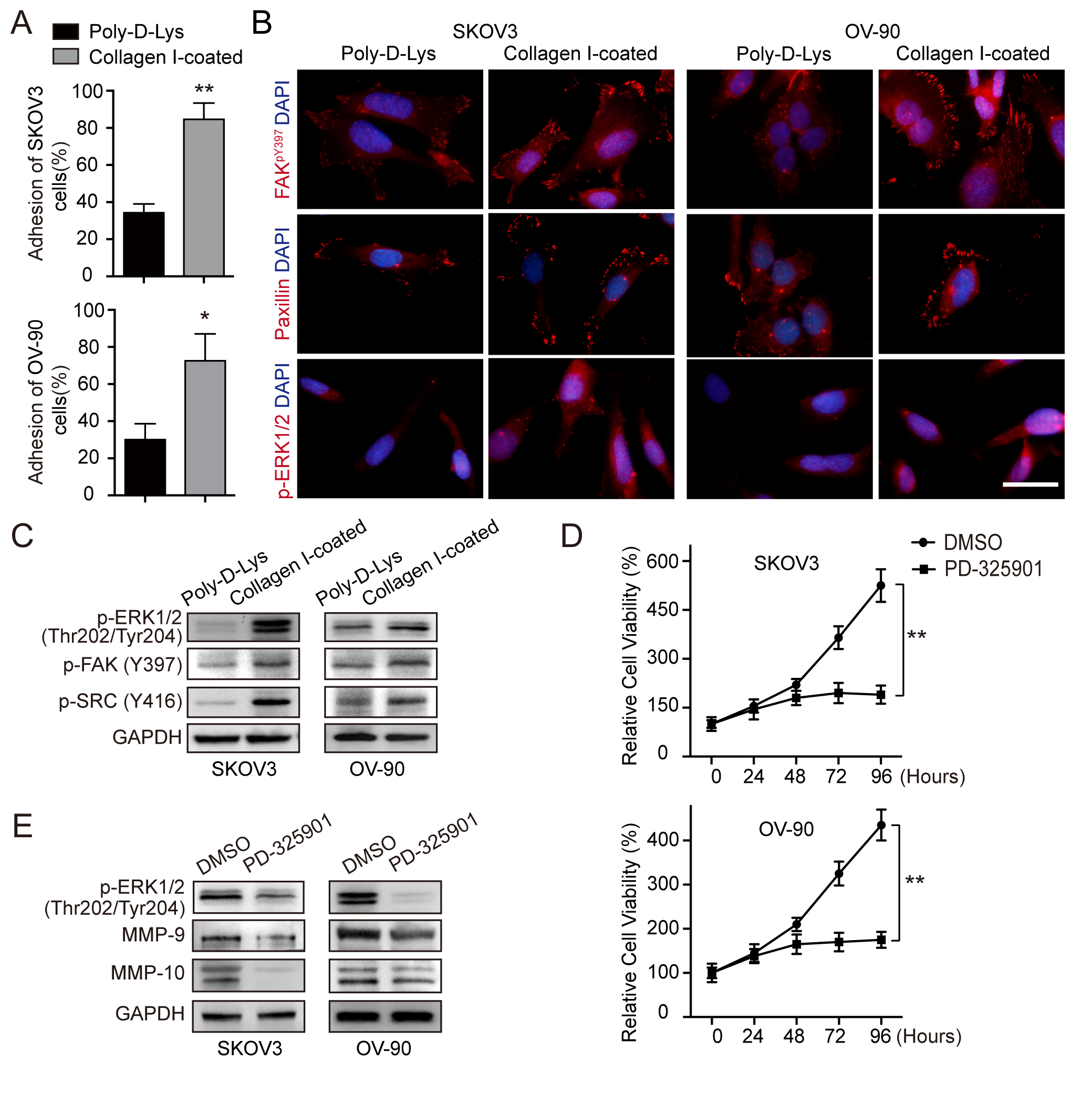

Supplement: Supplementary file 5 — Figure S4. Collagen signaling promotes adhesion and activates FAK-ERK linkage in ovarian cancer cells. (TIFF 1768 kb) [file 13046_2018_696_MOESM5_ESM.tif]
