# Supplementary material for: FAK-ERK activation in cell/matrix adhesion induced by the loss of apolipoprotein E stimulates the malignant progression of ovarian cancer
Source: J Exp Clin Cancer Res. 2018 Feb 20;37:32. doi: 10.1186/s13046-018-0696-4 (PMC5819228; doi:10.1186/s13046-018-0696-4)
Supplement: Supplementary file 2 — Table S1. The quantification of secreted factors in the cytokine profiling arrays. (DOC 210 kb) [file 13046_2018_696_MOESM2_ESM.doc]

| **Cytokine**  **(pg/ml)** | **WT** | ***APOE-*/*-*** | **Fold Change**  **(*APOE-*/*-* to WT)** |
| --- | --- | --- | --- |
| **Pro-MMP-9** | 764.8327452 | 47286.82752 | 61.82636376 |
| **MMP-10** | 3.321019754 | 70.66090835 | 21.27687083 |
| **E-Cadherin** | 4753.790893 | 12308.89736 | 2.589280352 |
| **ICAM-1 (CD54)** | 191.9408668 | 434.153753 | 2.2619141 |
| **MadCAM-1** | 11.28987696 | 22.98465684 | 2.035864246 |
| **MCP-5** | 622.2845502 | 137.8036931 | 0.221448039 |
| **bFGF** | 56.80864474 | 18.16867016 | 0.319822278 |
| **MMP-2** | 6306.995442 | 2090.438439 | 0.33144759 |
